# Supplementary material for: Chimerism Testing by Next Generation Sequencing for Detection of Engraftment and Early Disease Relapse in Allogeneic Hematopoietic Cell Transplantation and an Overview of NGS Chimerism Studies
Source: Int J Mol Sci. 2023 Jul 23;24(14):11814. doi: 10.3390/ijms241411814 (PMC10380370; doi:10.3390/ijms241411814)
Supplement: Supplementary file 1 [file ijms-24-11814-s001.zip › ijms-2503721-supplementary.pptx]

## Slide 1
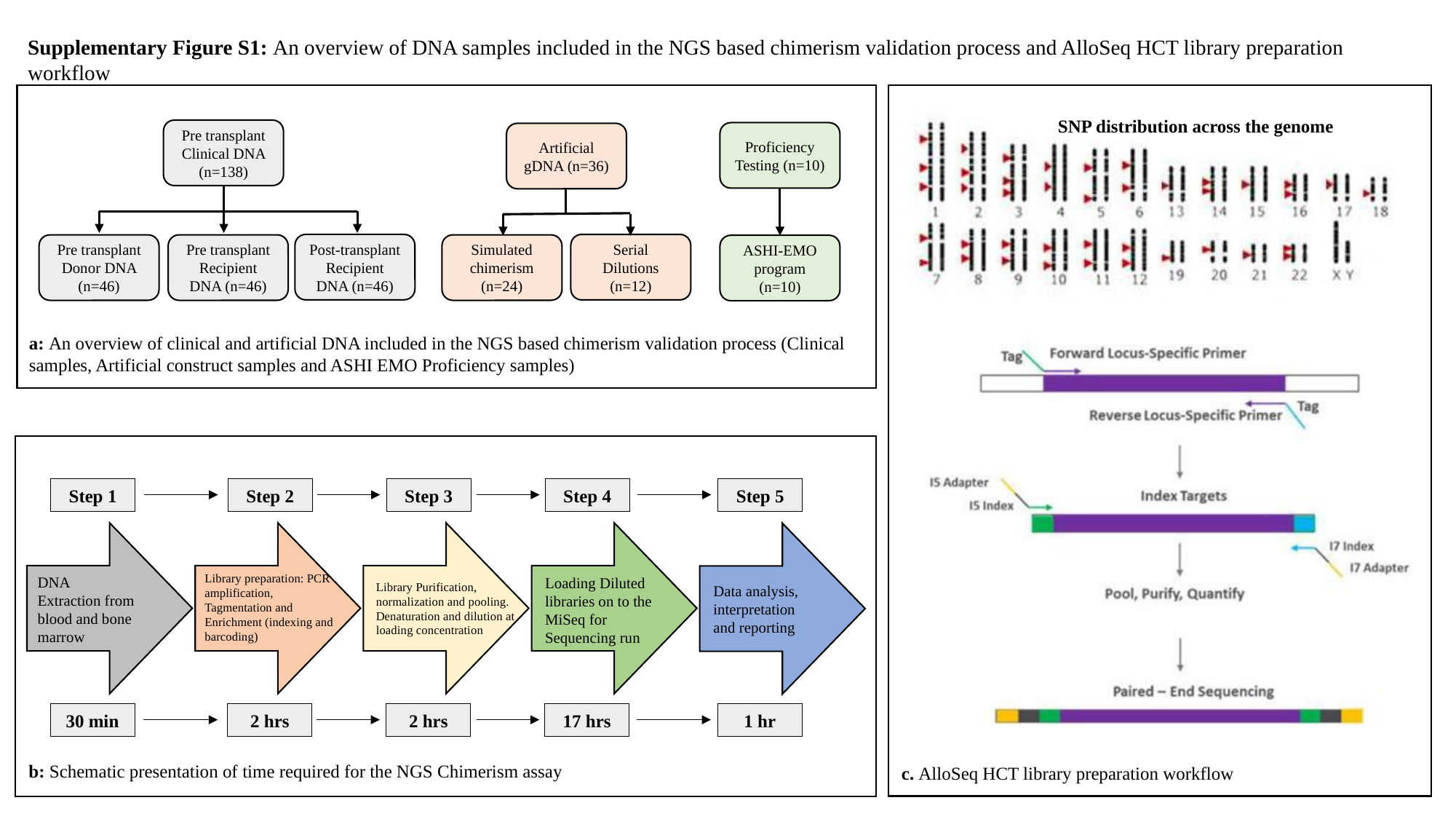

Supplementary Figure S1: An overview of DNA samples included in the NGS based chimerism validation process and AlloSeq HCT library preparation workflow
SNP distribution across the genome
Pre transplant Clinical DNA (n=138)
Proficiency Testing (n=10)
Artificial gDNA (n=36)
Post-transplant Recipient DNA (n=46)
Serial Dilutions (n=12)
Pre transplant Donor DNA (n=46)
Pre transplant Recipient DNA (n=46)
Simulated chimerism (n=24)
ASHI-EMO program (n=10)
a: An overview of clinical and artificial DNA included in the NGS based chimerism validation process (Clinical samples, Artificial construct samples and ASHI EMO Proficiency samples)
Step 5
Step 1
Step 2
Step 3
Step 4
Loading Diluted libraries on to the MiSeq for Sequencing run
Library preparation: PCR amplification, Tagmentation and Enrichment (indexing and barcoding)
Library Purification, normalization and pooling. Denaturation and dilution at loading concentration
DNA Extraction from blood and bone marrow
Data analysis, interpretation and reporting
1 hr
30 min
2 hrs
2 hrs
17 hrs
b: Schematic presentation of time required for the NGS Chimerism assay
c. AlloSeq HCT library preparation workflow
